# Blastic Plasmacytoid Dendritic Cell Neoplasm, from a Dermatological Point of View

**DOI:** 10.3390/ijms25137099

**Published:** 2024-06-28

**Authors:** Cosimo Di Raimondo, Flavia Lozzi, Pier Paolo Di Domenico, Claudia Paganini, Elena Campione, Marco Galluzzo, Luca Bianchi

**Affiliations:** 1 Dermatology Unit, Fondazione Policlinico Tor Vergata, 00133 Rome, Italyluca.bianchi@uniroma2.it (L.B.); 2 Department of Systems Medicine, University of Rome “Tor Vergata”, 00133 Rome, Italy

**Keywords:** blastic plasmacytoid dendritic cell neoplasm, BPDCN, skin cancer, cutaneous malignancies

## Abstract

Blastic plasmacytoid dendritic cell neoplasm (BPDCN) is an aggressive hematological malignancy derived from the precursors of plasmacytoid dendritic cells. Although disease awareness has increased over time, BPDCN represents a rare disease with an aggressive clinical course and a dismal prognosis. Due to the overlap in clinical and histological features with a large spectrum of inflammatory and neoplastic diseases, BPDCN is difficult to diagnose. Furthermore, given the rarity of the disease, treatment options for BPDCN are limited, sometimes changing by practitioner and hospitals. Treatment options range from conventional chemotherapy to the recently approved biologic agent tagraxofusp and stem cell transplantation. Therefore, a multidisciplinary approach with coordination among dermatologists, pathologists, and hematologists is ultimately imperative to reach the correct diagnosis and management of BPDCN.

## 1. Introduction

Blastic plasmacytoid dendritic cell neoplasm (BPDCN) is a rare and highly aggressive malignancy that origins from dendritic cells and involves skin, bone marrow, peripheral blood, lymphatic organs, and the central nervous system [1].

Due to the lack of data, the exact incidence of BPDCN is difficult to define, but it is estimated in 0.05 cases per 100,000 population. The incidence is significantly higher in Caucasian males, with a male:female ratio of 3.3:1 and a bimodal age distribution of <20 years and above 60 years [2,3]. In pediatric patients, the clinical characteristics do not differ from adults, yet in children, a better survival has been reported [4,5]. Currently, there is no evidence of environmental, inherited, or acquired genetic factors related to a higher risk of developing BPDCN. Approximately 10–20% of patients with BPDCN had a prior or concomitant diagnosis of acute myeloid leukemia (AML), chronic myeloid leukemia, chronic myelomonocytic leukemia (CML), or myelodysplastic syndromes [6,7,8,9].

BPDCN is characterized by an aggressive clinical course with a poor prognosis [10]. The 1-year, 3-year, and 5-year overall survival (OS) is estimated to be 90.7%, 83.7%, and 82.3%, respectively, in patients aged under 20, which drops to 53.1%, 27.7%, and 20.0% in patients aged over 60 years old [2,11].

This paper aims to review the clinical, histological, and therapeutic aspects of BPDCN from a dermatological perspective.

## 2. Dendritic Cells in the Skin

Dendritic cells (DCs) are bone-marrow-derived cells developing from lympho-myeloid lineages that coordinate innate and adaptive immune responses. Two different subsets of DC can be identified in the skin, namely, Langerhans cells (LCs), mainly localized in the epidermis, and interstitial DCs in the dermis [12]. In particular, the dermis hosts multiple DC subsets, including CD1a+ and CD14+ cells [13].

The function of skin DCs is critical in controlling immune responses in chronic inflammation, as well as maintaining immune homeostasis [12]. Their main biological role is to englobe disease-specific antigens and process and present them through MHC class I molecules to CD8+ T cells. Particularly, epidermal LCs are involved in priming and cross-priming naïve CD8+ T cells into potent cytotoxic T cells through the production of IL-15 [14,15]. Furthermore, many studies have demonstrated that LCs are also able to drive Th17/Th22 responses, suggesting a function in host defense against pathogens and tissue repair, as well as maintaining skin homeostasis through the expansion of skin Treg cells [12,16]. Both dermal CD14+ and CD1a+ DCs have common and unique features. In particular, CD14+ DCs are able, through the production of IL-12, IL-10 and TGF-β, to promote the activation of CD40-activated immature B cells into IgM-secreting plasma cells [17,18]. On the other hand, it has been demonstrated that dermal CD1a+ DCs have features of both LCs and dermal CD14+ DCs [12]. In fact, similar to LC’s, dermal CD1a+ DCs are able to produce IL-15 and have a key role in the activation of CD8+ naïve T cells into CTLs. Furthermore, as well as dermal CD14+, they are able to produce IL-8 [15,19].

## 3. Plasmacytoid Dendritic Cells in Dermatological Diseases

Plasmacytoid dendritic cells (PDCs) represent approximately 0.01% to 0.05% of peripheral blood mononuclear cells. They gather in inflammatory sites to enhance the inflammatory response [20].

PDCs have been now studied for decades due to their enigmatic features and functions [21]. In the late 1990s, the term “plasmacytoid dendritic cell” was coined to describe cells with a dichotomous paradigm [22]. In fact, plasmacytoid cells, in vitro, are able to produce type I interferons (IFN-I) and express low levels of major histocompatibility complex (MHC) class II, while after differentiation into DC, they lose their ability to produce IFN and they upregulate MHC class I and II [23,24,25,26]. Recent studies have demonstrated a common origin between PDCs and conventional DCs from a common DC precursor, regulated by Fms-like tyrosine kinase 3 ligand (Flt3L) [27]. Flt3L receptor (known as Flt3 or Flk-2) has homology with c-Kit/CD117, and it is highly expressed in hematopoietic progenitor cells [28,29]. Upon chemokine-driven stimulation, PDCs are recruited to different organs such as lymph nodes and tonsils and, much more rarely, spleen and mucosa associated lymphoid tissue [30,31]. PDCs are characterized by the expression of specific markers such as CD123 (IL-3 receptor α_chain) [32,33,34], CD68 [22], TCL1 (T-cell leukemia/lymphoma 1) [35], cutaneous lymphocyte-associated antigen (CLA)/HECA-452 [36], CD2-associated protein(CD2AP) [37], and blood dendritic cell antigen (BDCA)-2/CD303 [38].

The most specific markers for PDCs seem to be CD2AP and CD303, the latter having a key role in the antigen presentation to T cells [39]. On the contrary, PDCs are negative for lineage-specific markers for B cells, T cells, and NK cells, as well as myeloid or monocytic cells. A small amount of PDCs may express CD56 and some T cell markers such as CD2, CD5, and CD7 [40,41]. Despite being less than 0.1% of peripheral blood mononuclear cells, they represent the main source of type I interferon-α, therefore, being known also as interferon producing cells (IPCs) with the ability to differentiate into conventional dendritic cells (cDCs) in vitro [25]. The main mechanism of PDC activation that leads to massive IFN-I production seems to follow the signaling by endosomal TLRs through the adaptor MyD88 and transcription factor IRF7 [42].

While PDCs are rare in the skin in normal conditions, they can be detected in higher concentrations in various inflammatory cutaneous diseases. Farkas et al. demonstrated in 14 out of 15 tissue specimens of patients with lupus erythematosus (LE), but not in normal skin, the presence of PDCs, suggesting that PDCs are an important source of IFN-α/β in cutaneous LE lesions, thus, hypothesizing their role in the pathogenesis of cutaneous LE [43]. Moreover, Wollenberg et al. compared plasmacytoid dendritic cells of normal skin from healthy patients with that of lesional skin from patients with different inflammatory skin diseases [44]. They demonstrated high concentrations of PDCs in the lesional skin of patients with psoriasis and contact dermatitis, while in normal skin, PDCs were scarce or absent. In particular, some authors have demonstrated that in psoriatic patients with early stage disease, PDCs infiltrate lesions in high concentrations. Furthermore, blocking, in vitro, interferon-α production inhibits the development of psoriatic lesions [45]. In contrast, very few PDCs were described in patients with atopic dermatitis, exposing this subset of patients to a higher risk of viral infections [20]. Finally, Santoro et al. demonstrated a significant increase in CD123+/BDCA2+ PDCs in the epithelium and in the stroma of lichen planus biopsies compared to normal oral mucosa [43,44,46]. Moreover, many authors have demonstrated the key role of PDCs in antitumor immunity in various cutaneous malignancies. Increased PDCs have been described in melanoma, squamous cell carcinoma, basal cell carcinoma, and cutaneous T cell lymphoma [47,48] In the peritumoral area of melanoma patients, Vermi et al. demonstrated a large number of PDCs with a minor subset of mature CD1a(+) DCs. The lack of mature DCs and the predominancy of immature peritumoral dermal DCs may suggest the defective maturation of melanoma-associated DCs, resulting in a lack of T-cell priming, hence, leading to the proliferation of melanoma cells [49]. Notably, in melanoma, PDCs seem to enhance the tumor-specific T cell response; furthermore, PDC activation through a TLR9 agonist seems to be related to antitumor activity [50]. In basal cell carcinoma, melanoma in situ, and Bowen’s disease, authors have demonstrated how the application of topical imiquimod, a strong agonist of TLR7, leads to the recruitment of PDCs and over-expression of tumor-necrosis-factor-related apoptosis inducing ligand (TRAIL), thus resulting in cancer cell death [48,51,52].

Even though these data support the antitumor activity of PDCs mainly through the activation of TLR7 and TLR9, on the contrary, some authors have demonstrated how PDCs may also be involved in the suppression of host antitumor T-cell responses, mainly through the activation of regulatory T cells [53,54]. The study of PDC development facilitated the understanding of a highly aggressive leukemia type, the blastic plasmacytoid dendritic cell neoplasm (BPDCN), from its initial definition of a leukemic counterpart of PDCs to better diagnostic tools and potential therapeutic techniques aiming directly at PDCs [55,56,57].

## 4. Blastic Plasmacytoid Dendritic Cell Neoplasm (BPDCN)

BPDCN is an aggressive hematological malignancy derived from the precursors of plasmacytoid dendritic cells [11]. Due to the rarity of the disease and lack of large-scale studies, diagnosis and treatment procedures are still controversial. Therefore early diagnosis is fundamental to reach adequate treatment in a disease with a poor prognosis.

### 4.1. Genetics

Gene mutations in BPDCN involve different genes, mostly DNA methylation, histone modification, signal transduction, and splicing factors [58,59,60]. The most frequently mutated genes are TET2 and ASXL1, along with IKZF1, involved in BPDCN differentiation; RB1, ETV6, and NR3C1, the latter associated with glucocorticoid metabolism [59,61,62,63]. Yin et al. in a study on 50 BPDCN, detected mutations in 84% of patient, and 66% of patients had more than one mutation [60]. Furthermore, Sapienza et al. analyzed the gene expression profile of 25 patients with BPDCN, showing the aberrant activation of the NF-kB pathway and addressing it as a potential novel therapeutic target. They also identified, through FISH analysis, MYC rearrangement in approximately 40% and aberrations of IKZF1 with a controversial prognostic significance [59].

The rearrangement of MYC/8q24 or MYB/6q23) represents a unique pattern frequently detected in BPDCN. The activation of MYC induces the transcription of several target genes that regulate proliferation, division, metabolism, cell mobility, and apoptosis [64]. Cytogenetic abnormalities are detected in 57 to 75% of BPDCN patients, with several chromosomal losses or deletions. The most frequently reported chromosomal losses are 5q deletion, 12p deletion, 13q deletion or monosomy 13, 6q deletion, 15q deletion or monosomy 15, and monosomy 9, which compromise the normal function of the cells of origin of BPDCN [63,65,66]. Chromosomal microarrays confirm frequent losses of 9p21.3 (CDKN2A/CDKN2B), 12p13.2–p13.1 (CDKN1B, ETV6), 13q11–q21 (LATS2, RB1), 5q31 (NR3C1), or 7p12.2 (IKZF1) in patients with BPDCN [67,68]. Furthermore, a gene expression analysis of BPDCN revealed the upregulation of cyclin D1, a master regulator of cell cycle progression and BCL2 (Table 1) [69].

### 4.2. Clinical Features

**Figure 1:**
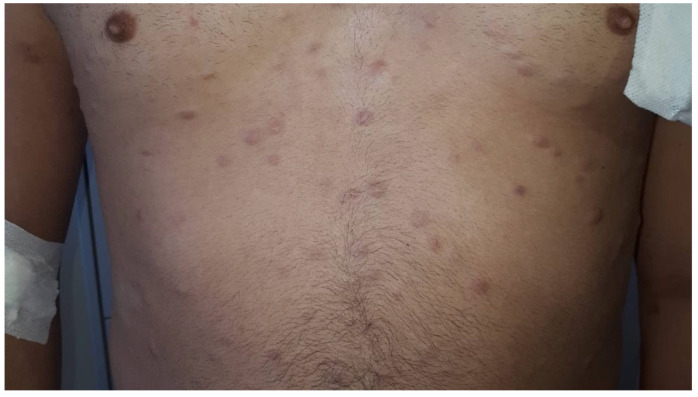
Disseminated nodules in a patient with BPDCN.

Due to the overlap in clinical and histological features with a large spectrum of inflammatory and neoplastic disease, BPDCN is difficult to diagnose. BPDCN typically appears on the skin with asymptomatic violaceous or brown patches, plaques or nodules, and bruise-like lesions, sometimes with ulceration [70,71,72]. Skin involvement ranges from one or a few nodules localized in a single cutaneous district to a broad cutaneous dissemination (Figure 1) [73]. In a retrospective analysis of 90 patients with BPDCN, Julia et al. have demonstrated that disseminated cutaneous lesions represented only 15% of patients [70]. The most commonly involved sites are head, upper trunk, and upper extremities, followed by lower trunk and extremities [73]. Dermoscopic features range from purple structureless areas with a white halo to purple structureless areas alternated with homogeneous bluish-white areas. The purple structureless areas seem to be correlated with the presence of a dense infiltrate of neoplastic cells in the dermis and intratumoral hemorrhage; the white halos represent the dermal grenz zone; and the homogeneous bluish-white areas are consistent with fibrosis in the papillary dermis [74,75,76]. More than 60% of patients have bone marrow involvement and more than 20% of patients have a prior or concomitant myeloid malignancy [77,78,79]. Several studies have demonstrated that patients with isolated cutaneous lesions have a better prognosis than those with widespread lesions [80]. On the contrary, Julia et al. have demonstrated that although patients with disseminated skin involvement at diagnosis are more likely to have detectable systemic disease, the extent of skin disease does not seem to correlate with survival [70]. Mucosal localizations have rarely been reported in the literature, mainly with pharynx and nasal involvement [81]. Yu et al. reported a case of a 67 year-old female with primary nasal BPDCN of the nasal cavity without cutaneous involvement [82]. In the differential diagnosis of primary nasal BPDCN, many tumors need to be considered, namely, melanoma, mesenchymal chondrosarcoma, squamous cell carcinoma, olfactory neuroblastoma, and pituitary adenoma [83].

### 4.3. Differential Diagnosis

BPDCN can mimic various neoplastic and non-neoplastic conditions, as demonstrated in numerous reports in the literature. An increase in PDCs in the skin has been described in several inflammatory cutaneous conditions such as systemic lupus erythematosus, psoriasis, lichen planus, and contact dermatitis [21]. Dermatologists need to be aware of atypical clinical presentations of BPDCN in order to prompt early diagnosis and avoid misdiagnoses. Fay et al. reported a case of BPDCN mimicking dermatomyositis, with purple infiltrative thin plaques over the bilateral eyelids, mimicking heliotrope rash, and violaceous macules and patches on the trunk [84]. Further atypical presentations are represented by erythematous patches, hyperemic nodules, and plaques on the trunk, face, arms, and thighs mimicking cutaneous lupus erythematosus [85]. BPDCN may present also with a solitary skin purpuric lesion resembling traumatic purpura [86]. Among neoplastic diseases, the most frequent and challenging differential diagnosis for BPDCN is with acute myeloid leukemia (AML)/leukemia cutis. In fact, BPDCN-circulating blasts can morphologically resemble myeloid blasts; furthermore, AML shares some immunophenotyping with BPDCN, including CD123, CD56, and sometimes CD33 [87].

Immunophenotypic overlap may be seen also with NK/T-cell leukemia/lymphoma being positive for CD2 and CD56 but associated with Epstein-Barr virus infection and sometimes positive for EBER [3]. Additional differential diagnoses include hematological neoplasms with blastic morphology, such as the blastoid variant of mantle cell lymphoma and lymphoblastic lymphoma of B-cell or T-cell origin [88]. Finally, Kaposi sarcoma, angiosarcoma, and Merkel cell carcinoma represent common differential diagnoses (Table 2) [73,89].

### 4.4. Diagnosis

As was already mentioned above, BPDCN is difficult to diagnose, in particular in those patients who have nonspecific cutaneous lesions. The diagnosis of BPDCN is usually made through a biopsy of the skin that represents the most common site of disease (Table 3). Therefore, wide deep dermis skin biopsies, along with immunophenotyping, cytologic evaluation, and flow cytometry, are essential to reach a final diagnosis. It is important for dermatologists to address BPDCN to the pathologist as a probable clinical differential diagnosis, both for the need of rare immunohistochemical (IHC) stains, not always available, and also for the frequent histological overlap with other cutaneous diseases such as, for instance, leukemia cutis. Although it seems to differ amongst patients, a common phenotypic presentation includes cells that are negative for CD3, CD13, CD16, CD20, lysozyme, and MPO but positive for CD4, CD56, CD123, CD303, and TCL1A; CD7 and CD3 are also frequently expressed [11]. From 10 to 80% of tumor cells express terminal deoxynucleotidyl transferase, a nuclear enzyme that plays a key role in the development and variation of the immune system [90,91]. Cytologic evaluation usually shows dispersed dermal and subcutaneous infiltrates of atypical medium-sized blastic cells, sparing epidermis and adnexal, mainly with round to irregular nuclei, scarce pink cytoplasm, and faint cell distinction. Sometimes occasional cells show eccentric nuclei with scanty cytoplasm [92,93]. The Ki-67 rate ranges from 20% to 80% [94]. In patients with cutaneous and subcutaneous involvement, the function of imaging is limited. CT findings usually reveal nodular or plaque-like skin thickening, while PET/CT or magnetic resonance imaging (MRI) can reveal lymph nodes or central nervous system involvement [95,96,97].

## 5. Pediatric BPDCN

The data on pediatric BPDCN are limited due to its rarity; therefore, the exact incidence of BPDCN in children is still unknown [98]. Abnormalities in the MYB locus, a DNA-binding transcription factor and one of the key regulators of vertebrate hematopoiesis, seem to be higher in children than in adults. Thus, the proto-oncogene is identified as a potential diagnostic marker and molecular therapeutic target in pediatric BPDCN [99,100,101]. In the largest systematic literature review, among 74 pediatric cases, Kim. et al. showed no differences in clinical presentation among children compared to adults. On the contrary, age was shown to be an independent prognostic factor predictive of better prognosis and advantages in terms of initial response to therapy, likelihood of relapse, and overall survival. Children with BPDCN had a significantly higher CR rate [86% vs. 52%, *p* < 0.01], were less likely to relapse (27% vs. 57%, *p* < 0.01), and were more likely to be alive and disease-free at follow-up (68% vs. 27%, *p* < 0.01) compared to adults [5]. Jegalian et al., in a review of 20 pediatric cases, reported that 24% of patients lacked cutaneous involvement, which is a slightly higher percentage than described in adult patients [3] The prognosis of pediatric BPDCN is generally more favorable than adults [102]. A case study conducted in patients with ages ranging from less than 1 year to 18 years demonstrated that pediatric BPDCN is clinically less aggressive and frequently associated with a more favorable outcome when treated with high-risk acute lymphoblastic leukemia (ALL) chemotherapy and central nervous system (CNS) prophylaxis [3]. Furthermore, Jegalian et al. reported a 72% survival rate in a study of 25 pediatric patients receiving chemotherapy [3].

## 6. Treatment

Given the rarity of the disease, treatment options for BPDCN are limited, sometimes changing by practitioner and center. Treatment options range from conventional chemotherapy to the recently approved biologic agent tagraxofusp and stem cell transplantation. Pre-treatment evaluation should include complete blood counts, liver and kidney function tests, lactate dehydrogenase, hepatitis B, and HIV. While skin lesions usually need more time to resolve compared to those involving blood, bone marrow, lymph nodes, and central nervous system, they can be identified as an easily reachable supplementary parameter of the treatment response [103]. To date, the only potentially curative treatment for BPDCN remains the allogeneic hematopoietic stem cell transplantation (HSCT) [104]. Traditionally, three main conventional chemotherapy regimens are usually used for BPDCN: acute myeloid leukemia (AML) regimens with idarubicin, cytarabine and etoposide or with mitoxantrone, cytarabine and etoposide; acute lymphoblastic leukemia (ALL) regimens with hyperfractionated cyclophosphamide, vincristine, and Adriamycin; and lymphoma regimens with CHOP (cyclophosphamide, adriamycin, vincristine, prednisone) or CHOP-like regimens (CHOP + etoposide) [105]. Even so, despite being sensitive to first line chemotherapy, with a complete remission rate of 53% to 89%, more than 60% of patients relapse, with a median survival of 12 to 18 months [106]. In 2018, tagraxofusp a CD123-based fusion protein SL-401, was approved by the Food and Drug Administration (FDA) as a treatment for all patients with BPDCN aged ≥2 years that radically changed the treatment landscape for patients with this rare and aggressive neoplasm [107] (Table 4).

The same drug has also been approved in the EU, but exclusively for the treatment of first-line adult patients, by the European Medicines Agency. Tagraxofusp consists of interleukin-3 linked to the C-terminus of the truncated diphtheria toxin that causes cell death by inhibiting cellular protein translocation by adenosine 5′-diphosphate ribosylation of eukaryotic elongation factor 2 [108,109]. The pivotal study was an open-label, multicohort study, where 47 patients with untreated or relapsed BPDCN received an intravenous infusion of tagraxofusp at a dose of 7 μg or 12 μg per kilogram of body weight on days 1 to 5 of each 21-day cycle, with an overall response rate of 90%. Among all patients, 45% underwent stem-cell transplantation with survival rates at 18 and 24 months of 59% and 52%, respectively [107,110]. A recent real-world analysis of five male patients with BPDCN who received tagraxofusp as first line therapy showed response in three patients out of five with two complete response (CR) and one partial response (PR) [111]. In thirteen case reports of tagraxofusp first-line treatment for BPDCN, eight patients reached a CR (61.5%), two a PR (15.4%) and two showed stable disease, with a median response duration of 9 months. In both case reports and clinical trials, common serious adverse events from tagraxofusp included capillary leak syndrome, hepatic dysfunction, and thrombocytopenia [111,112,113,114,115,116,117,118,119,120,121]. Capillary leak syndrome represents a specific toxicity related to tagraxofusp that usually is associated with edema, weight gain, hypoalbuminemia, and cardiovascular involvement, which can be rapidly deadly if not diagnosed and managed correctly [109]. On the other hand, liver dysfunction and thrombocytopenia are usually mild and reversible. Given the efficacy of Tagraxofusp as a single agent, it is now being investigated in association with other drugs. Preliminary data from the phase 1b trial evaluating tagraxofusp with azacitidine or with azacitidine plus venetoclax demonstrated that in two of the three patients with relapsed BPDCN, there was a complete remission [122].

To date, allogeneic hematopoietic stem cell transplantation (allo-HSCT) represents the only potentially curative option for BPDCN. Different studies have shown that consolidation therapy with HSCT after disease complete remission increases overall survival, allo-HSCT being more effective in terms of higher remission rate and a lower relapse rate compared to auto-HSC [123,124,125]. In a report of 164 patients with BPDCN who underwent allo-HCT, the 5-year overall survival (OS) and disease-free survival (DFS) rates were 51.2% and 44.4%.

On multivariate analysis, the authors concluded that an age of ≥60 years together with remission status at time of allo-HCT was predictive of inferior OS. On the other hand, myeloablative conditioning with total body irradiation was related to a better disease-free survival and lower risk of relapse. These data confirm the efficacy of allo-HCT leading to durable remissions and long-term survival, especially in young patients with BPDCN [126]. Hirner et al. described their clinical experience with total skin electron beam therapy as part of transplant conditioning with the aim of inducing remission in the further subclinical disease burden at the time of SCT [73].

Given the upregulation of cyclin D1, Montero et al. demonstrated that primary BPDCN cells depend on BCL2 and are sensitive to BCL2 inhibition, both in vitro and in vivo [127]. Therefore, an ongoing single-arm phase 1 trial (NCT03485547) is investigating venetoclax, a selective small-molecule inhibitor of BCL2 in combination with other molecules [128]. Moreover, Pemmaraju et al. investigated the role of venetoclax in combination with hypomethylators and cytotoxic chemotherapy. They showed that three out of three patients who received hyper-CVAD (hyperfractionated cyclophosphamide, vincristine, doxorubicin, and dexamethasone) plus venetoclax had complete remission with no severe adverse events. These preliminary data show the feasibility of BCL2 inhibitors in combination with targeted or cytotoxic therapies in blastic plasmacytoid dendritic-cell neoplasm [129]. Given the overexpression of CD123 in BPDCN cells, Pemmaraju et al. demonstrated the efficacy of antibody–drug against CD123, IMGN632 in 23 patients heavily pretreated with relapsed or refractory BPDCN, thus, representing a good option in patients who fail tagraxofusp [129]. Furthermore, flotetuzumab, a bispecific antibody targeting CD123 is currently under investigation in patients who are refractory or had relapsed after tagraxofusp first-line therapy [122].

## 7. Conclusions

Although disease awareness has increased over time and the always growing knowledge of BPDCN in recent years has helped with developing a diagnostic work-up, there is still a high risk of misdiagnosis. Richard at al. have recently analyzed the journey of patients with skin diseases through healthcare in Europe, on a total of 44,689 individuals from 27 European countries. The authors have demonstrated that in approximately 40% of participants, the diagnosis of skin cancer was made after consultation with at least one other medical specialist. Diagnostic procedures, such as biopsy or other techniques, were mandatory to make the final diagnosis in 84.6% of patients with skin cancer. [130]. This analysis addresses the needs of the patient with skin cancer, emphasizing the importance of reducing diagnostic delay in neoplastic cutaneous diseases such as BPDCN; thus, improving outcomes and reducing health care expenses [131]. Delay in the correct diagnosis of cutaneous BPDCN may lead to disease spread and progression to systemic involvement, thus, requiring dermatologists to provide a prompt and correct diagnosis. It is important to suspect BPDCN and recognize early skin lesions in order to perform a skin biopsy promptly, with a close collaboration of the clinician with the pathologist [118]. Furthermore, with new therapies arising in the landscape of treatment and showing promising results in terms of efficacy and safety in BPDCN, combining these drugs with allo-HCT will be critical to further improve the outcomes of patients with BPDCN. The choice of the most appropriate treatment may depend not only on the clinical manifestations and the extent of the disease but also on the availability of different drugs according to different institutions and countries. Therefore, a multidisciplinary approach with coordination among dermatologists, pathologists, and hematologists is ultimately imperative to reach a correct diagnosis and management of BPDCN.

## Figures and Tables

**Table 1:** Frequent genetic mutations in BPDCN.

Mutated genes [58,59,60,61,62,63]	TET2 ASXL1 IKZF1 RB1 ETV6 NR3C1 MYC
Chromosomal losses [64,65,66]	5q deletion 12p deletion, 13q deletion or monosomy 13 6q deletion 15q deletion or monosomy 15 monosomy 9

**Table 2:** Differential diagnosis of BPDCN with cutaneous involvement [21,84,85,86].

Differential Diagnosis	
Non Neoplastic Diseases	Neoplastic Diseases
Systemic lupus erythematosus	AML/leukemia cutis
Psoriasis	NK/T-cell leukemia/lymphoma
Lichen planus	hematological neoplasms with blastic morphology
Contact dermatitis	Kaposi sarcoma
Traumatic purpura	angiosarcoma
	Merkel cell carcinoma

**Table 3:** Diagnostic clues of BPDCN [11].

Clinical features	Violaceous or brown patches, Plaques, nodules Bruise-like lesions Ulceration Head, upper trunk, and upper extremities
Dermoscopic features	Purple structureless areas White halo Homogeneous bluish-white areas
Histologic features	dermal and subcutaneous blastic cells sparing epidermis and adnexal round to irregular nuclei scarce pink cytoplasm
Immunophenotype	CD3, CD4, CD7, CD56, CD123, CD303, TCL1A

**Table 4:** Treatment options for BPDCN [107,108,109].

Acute myeloid leukemia regimen	idarubicin cytarabine and etoposide cytarabine and mitoxantrone
Acute lymphoblastic leukemia regimen	hyperfractionated cyclophosphamide, vincristine, and adriamycin
Lymphoma regimens	CHOP CHOP + etoposide
Biologic agent	Tagraxofusp
Allo-HSCT	
